# An investigation into the effect of evening primrose in dilatation of cervix and pain during and after hysterosalpingography

**DOI:** 10.25122/jml-2019-0021

**Published:** 2019

**Authors:** Shahrzad Shahnam Nia, Fatemeh Safi, Maryam Shoukrpour, Alireza Kamali

**Affiliations:** 1. Department of Radiology, Arak University of Medical Sciences, Arak, Iran; 2. Department of Gynecology, Arak University of Medical Sciences, Arak, Iran; 3. Department of Anesthesiology and Critical Care, Arak University of Medical Sciences, Arak, Iran

**Keywords:** Pain, Cervical length, Evening primrose, Hysterosalpingography

## Abstract

Hysterosalpingography is one of the essential diagnostic methods for examining women who have difficulty becoming pregnant. This procedure is somehow invasive and is associated with numerous complications such as allergic sensitivity, pain, abdominal cramps and shock. Therefore, this study aimed to investigate the effect of evening primrose on cervical length and pain during and after hysterosalpingography. In this double-blind clinical trial, 66 candidates for hysterosalpingography were randomly divided into two groups. A group received 1000 mg of evening primrose orally for two days prior to hysterosalpingography, while the control group received a placebo drug similar in size to evening primrose three days prior to hysterosalpingography. The pain level was recorded based on the Visual Analogue Scale (VAS), during tenaculum placement but also immediately and four hours after hysterosalpingography. Finally, the data were analyzed using SPSS (version 20). There was a significant difference between the two groups in terms of pain during insertion of speculum and injection of the contrast medium (p <0.05). Less pain was reported in the evening primrose group compared to placebo. There was no significant difference between the two groups in terms of the length and diameter of the cervix (p <0.05). Given the fact that it is a medicinal plant with no complications and can reduce pain during speculum insertion and during contrast medium injection, evening primrose seems to be a good drug for managing pain during hysterosalpingography.

## Introduction

Infertility is one of the most common problems in women. This complication involves a heterogeneous group of patients, and infertility-related assessments are required to investigate its causes. Hysterosalpingography (HSG) is one of the important diagnostic methods for assessing the openness of the uterus and uterine tubes [1]. Uterine cavity abnormalities account for 10-15% of infertility causes in couples [2]. Uterine abnormalities are found in approximately 34-62% of infertile women; given the high prevalence of uterine abnormalities, uterine cavity examination is performed in the initial assessment of infertility [3]. Hysterosalpingography uses a real-time form of x-ray called fluoroscopy to examine the uterus and fallopian tubes of a woman who is having difficulty becoming pregnant. It is also used to investigate miscarriages resulting from abnormalities within the uterus and to determine the presence and severity of tumor masses, adhesions and uterine fibroids. Hysterosalpingography can occasionally open blocked fallopian tubes to allow the patient to become pregnant afterward. This method has been used since 1922 as the first and easiest diagnostic test for uterine tubes abnormalities [4]. As a low-priced and simple method, hysterosalpingography is used to detect these abnormalities. However, it is an invasive method and is associated with several complications such as increased sensitivity, pain, abdominal cramps and even shock [5, 6]. In 2004, the National Institute for Health and Care Excellence (NICE) issued the guidelines for hysterosalpingography and introduced it as a useful tool to screen for fallopian tube occlusion [7]. Szymusik et al. argued that the occlusion of fallopian tubes and anomalies of the uterine cavity were affecting pain intensity during hysterosalpingography, but did not affect the previous delivery type and parity [8]. A significant number of people are seeking alternative medicine for the treatment of various diseases. Hence, the use of complementary and alternative medicine is constantly increasing [9]. As an alternative treatment, herbal medicines may have a relative superiority and improve drug delivery [10]. Evening primrose, also known as Oenothera biennis, is an herbal medicine belonging to the Onagraceae family. There is usually a central stem with alternate leaves, but sometimes there will be multiple stems in open areas, creating a bushy appearance. The stems are light green or red and are covered with white hairs. They prefer full sun, average moisture, and somewhat sandy soil, but other growing conditions are acceptable. This plant forms a rosette during the first year but becomes tall during the second year, at which time it flowers, sets seed, and dies. Common Evening Primrose is easy to grow but often becomes rather unsightly as the season progresses. There are about 145 species in the genus *Oenothera* L., growing in the temperate and tropical climate zones of North and South America. Some species have adapted to new areas, inhabiting the countries of the European continent, and about 70 species are now present in Europe. The most numerous species in the *Oenothera* L. family is *Oenothera biennis*, which also has the best-studied biological activity. It has been indicated that *Oenothera biennis* is beneficial in the treatment of many diseases. Therefore, research is ongoing to determine the chemical composition of these plants and how it relates to the biological activity of evening primrose. This research mainly concerns extracts from various parts of evening primrose (e.g., the leaves, stems, and seeds) [11]. Evening primrose oil can reduce menstrual pain, treat diabetes mellitus, and reduce cholesterol [12]. Many women find that evening primrose oil offers relief from common symptoms of premenstrual syndrome (PMS) and premenstrual dysphoric disorder (PMDD), particularly mood swings, breast tenderness and menstrual cramps. It is thought that the ability of gamma-linolenic acid (GLA) to regulate prostaglandins helps to reduce the body’s inflammatory response to hormone fluctuations that occur around menstruation. This extract is rich in essential fatty acids and contains 7-14% gamma-linoleic acid. The evening primrose extract has also been used in the treatment of mastalgia, due to its anti-inflammatory effects [13, 14]. Alvandipour et al. [15] and Pruthi et al. [16] argued that vitamin E and evening primrose extracts have similar therapeutic effects for the treatment of breast pain. This result can be indicative of anti-inflammatory and analgesic effects of evening primrose’s extract. It can be used to manage hysterosalpingography-related pain. Given the fact that herbal medicines are popular, studies in the past have focused on the treatment of hysterosalpingography-related pain by chemical drugs. However, limited studies have focused on the management of hysterosalpingography-related pain employing evening primrose. Therefore, this study aimed to investigate the effects of evening primrose on cervical length and pain during and after hysterosalpingography.

## Materials and Methods

In this double-blind clinical trial, 66 candidates for hysterosalpingography were randomly divided into two groups. A group received 1000 mg of evening primrose orally two days prior to hysterosalpingography, while the control group received a placebo drug similar in size to evening primrose three days prior to hysterosalpingography.

### Inclusion criteria

Infertility; patients aged 19 to 42 years; candidate for hysterosalpingography.

### Exclusion criteria

Severe dysmenorrhea; pelvic infections; drug sensitivity; duration of HSG for more than 45 minutes; hemodynamic instability during HSG; unwillingness to participate in the study; vaginal delivery; secondary infertility.

A demographic questionnaire including age, parity, history of dysmenorrhea (mild, moderate, severe), history of hysterosalpingography in the past, and history of endometrial biopsy was recorded for all patients. Patients were randomly divided into two groups. A group received 1000 mg of evening primrose orally for two days prior to hysterosalpingography, while the control group received a placebo drug similar in size to evening primrose three days prior to hysterosalpingography. Afterward, all patients underwent hysterosalpingography. A metal cannula was gently inserted into the external cervical canal, the cannula and tenaculum were secured together, and the speculum was removed. Once the cervix was detected, betadine solution was used to probe the area. The anterior and posterior lips of the cervix were held with the tenaculum, and the cannula was inserted through the cervical canal. The speculum was then removed.

The patient was asked to assess pain intensity using the visual analogue scale. A Visual Analogue Scale (VAS) is a measurement instrument that tries to measure a characteristic or attitude that is believed to range across a continuum of values and cannot easily be directly measured. It is often used in epidemiology and clinical research to measure the intensity or frequency of various symptoms. After insertion into the vagina, the tenaculum allows healthcare professionals to grasp the cervical tissue, pull and stabilize the external part of the cervix. Tenaculum consists of a slender sharp-pointed hook, and it causes pain and bleeding. Based on HSG findings, the presence or absence of spasm of the fallopian tubes was determined. Having the patient empty her bladder prior to positioning may ease discomfort from the speculum, which should be warmed prior to insertion. Supporting the patient’s hips on a short stack of towels or an overturned bedpan often aids in the visualization of the cervix.

As with any pelvic examination, the operator should attempt to drape as much of the patient’s lower body as possible, have a female chaperone, and explain to the patient each step of the procedure. At the end of the procedure, all instruments were removed, and the patient was observed and monitored for half an hour in the clinic. The pain level was recorded based on the Visual Analogue Scale (VAS) during tenaculum placement but also immediately and four hours after hysterosalpingography. In this scale, zero and ten represent the lowest and the highest amount of pain, respectively. Then, the pain was compared in the two groups. Pain intensity was compared between the two groups at the time of fallopian tube and abnormalities in the uterine cavity. Accordingly, the anomalies of the uterine cavity were also compared.

Moreover, vasovagal responses were recorded and compared in patients of both groups. The vasovagal response is the development of inappropriate cardiac slowing and arteriolar dilatation. Vasovagal responses reflect autonomic neural changes: bradycardia results from sudden augmentation of efferent vagal activity, and hypotension results from sudden reduction or cessation of sympathetic activity and relaxation of arterial resistance vessels. Two different neural pathways are thought to be involved, one originating in the hypothalamus, the other in the heart.

It should be noted that neither the radiology assistants nor the patients had access to the data. Finally, the data were analyzed using SPSS. The ethical committee code is: IR.ARAKMU.REC.1396.42

## Results

This double-blind clinical trial included 66 patients undergoing hysterosalpingography in Radiology Clinic of Arak University of Medical Sciences. The patients were randomly divided into two groups. The minimum and the maximum was 19 years and 42 years, respectively. The mean age of the patients was 27.74 ± 4.77 years. The duration of infertility was 2.65 ± 3.17 years. Accordingly, the duration of infertility in the evening primrose group and the placebo group was 3.36 ± 4.9 and 2. 04 ± 1.71, respectively. There was no significant difference between the two groups (p = 0.20). According to the results, there was no significant difference between the two groups in terms of age (p = 0.939). The results showed that there was no significant difference between the two groups in terms of parity, dysmenorrhea, history of dyspareunia, history of hysterosalpingography and history of endometrial biopsy (p> 0.05) (Table 1).

**Table 1:** Comparison of the percentage and frequency of qualitative variables in the evening primrose and placebo groups

Group	Qualitative variable	Evening primrose	Placebo	p-value
Parity	Nulliparous	32 (96.96)	33 (100)	0.5
	Multi-parity	1 (3.03)	0 (0)	
Dysmenorrhea	No	0 (0)	5 (15.15)	0.120
	Mild	21 (61.63)	20 (60.60)	
	Medium	9 (27.27)	6 (18.18)	
	Severe	3 (9.09)	2 (6.06)	
History of dyspareunia	Yes	2 (6.06)	3 (9.09)	0.5
	No	31 (93.93)	30 (90.90)	
History of hysterosalpingography	Yes	3 (9.09)	1 (3.03)	0.307
	No	30 (90.90)	32 (96.96)	
History of endometrial biopsy	Yes	0 (0)	0 (0)	>0.05
	No	33 (100)	33 (100)	

The results showed that there was no significant difference between the two groups in terms of vasovagal responses, fallopian tube spasm and uterine cavity anomalies (Table 2).

**Table 2:** Comparison of percentage and frequency of complications during hysterosalpingography in the evening primrose and placebo groups

Group	Variable	Evening primrose	Placebo	p-value
Vasovagal responses	Yes	1 (3.03)	1 (3.03)	0.754
	No	32 (96.96)	32 (96.96)	
Tubal spasm	Yes	0 (0)	0 (0)	>0.05
	No	33 (100)	33 (100)	
Uterine cavity anomalies	No	29 (87.87)	30 (90.90)	0.503
	Polyp	1 (3.03)	1 (3.03)	
	*Submucosal fibroid*	1 (3.03)	2 (6.06)	
	Septum	0 (0)	0 (0)	
	Endometrial adhesion	2 (6.06)	0 (0)	
	Other	0 (0)	0 (0)	

**Figure 1:**
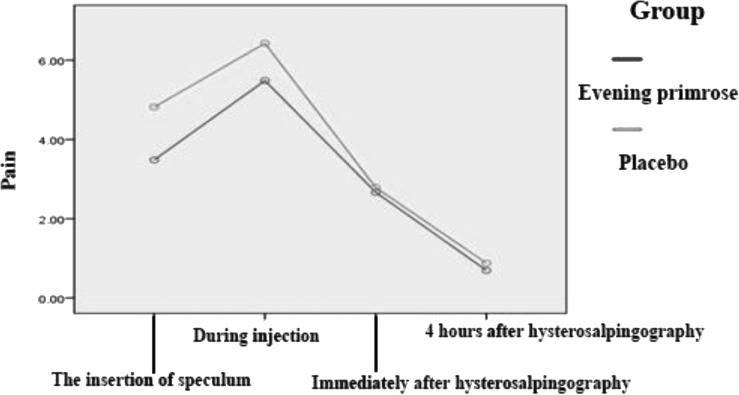
Comparison of pain at different times in the evening primrose and placebo group

According to Table 3, there was a significant difference between the two groups in terms of pain during speculum insertion (p = 0.033). There was also a significant difference between the two groups in terms of pain during injection (p = 0.023). Less pain was reported in the evening primrose group compared to placebo. There was no significant difference between the two groups in terms of pain immediately after the procedure (P = 0.842). Also, there was no significant difference between the two groups in terms of pain at four hours after hysterosalpingography (p = 0.576) (Table 3). As shown in Figure 1, the perceived pain was lower in the evening primrose group compared to the placebo group during speculum insertion and contrast medium injection.

**Table 3:** Comparison of percentage and frequency of pain in the evening primrose and placebo group

Group Variable	Evening primrose Mean - SD	Placebo Mean - SD	p-value
During the insertion of speculum	3.48±2.04	4.81±2.84	0.033
During contrast medium injection	2.30 ± 5.48	6.42±3.17	0.023
Immediately after hysterosalpingography	2.69±2.39	2.78±2.53	0.842
4 hours after hysterosalpingography	0.677±1.1	0.787 ±1.55	0.576

According to Table 4, there was no significant difference between the two groups neither in terms of length and diameter of the cervix in (p> 0.05), nor in terms of hysterosalpingography duration (p = 0.918).

**Table 4:** Comparison of mean and standard deviation of cervical profile in the evening primrose and placebo group

Group Cervix	Evening primrose Mean - SD	Placebo Mean - SD	p-value
The length of the cervix	1.33±0.32	1.28±0.27	0.805
The diameter of the cervix	0.398±0.188	0.158±0.124	0.507
Duration of hysterosalpingography	9.58±2.63	9.48±4.30	0.918

## Discussion

As a low-priced and simple method, hystero­salpingography is used to detect these abnormalities. This invasive method is associated with several complications such as increased sensitivity, pain, abdominal cramp and even shock [17]. Recent studies have focused on the treatment of hysterosalpingography-related pain by using chemical drugs. However, limited studies have focused on the management of hysterosalpingography-related pain utilizing evening primrose. Therefore, this study aimed to investigate the effect of evening primrose on cervical length and pain during and after hysterosalpingography. The results showed that the minimum and maximum age of the patients was 19 years and 42 years, respectively. Accordingly, the mean age of the patients was 27.74 ± 4.77 years. The mean infertility period was 2.65 ± 3.17 years, which was 3.9 ± 4.9 years and 2.4 ± 1.71 years in the evening primrose and the placebo group, respectively. There was no significant difference between the two groups in this regard (p=0.120). Also, there was no significant difference in terms of age, parity, dysmenorrhea, history of dyspareunia, history of hysterosalpingography and history of endometrial biopsy (p <0.05). There was no significant difference between the two groups in terms of vasovagal responses, fallopian tube spasm, and anomalies of the uterine cavity (P> 0.05). However, there was a significant difference between the two groups in terms of pain during speculum insertion, meaning that the patients in the evening primrose group reported less pain compared to the placebo group (p = 0.033). Additionally, there was a significant difference between the two groups in terms of pain during injection, meaning that pain was less reported in the evening primrose group compared to placebo (p = 0.023). There was no significant difference between the two groups in terms of pain immediately and four hours after hysterosalpingography, neither in terms of length and diameter of the cervix (p <0.05). Also, there was no significant difference between the two groups in terms of the duration of hysterosalpingography (p = 0.918).

Szymusik et al. investigated the factors influencing pain during hysterosalpingography. In their study, 89 patients were assigned to the ketoprofen group and received 0.1 g of ketoprofen. Likewise, 89 patients were assigned to the metamizole group and received 2.5 g of metamizole. The pain level was measured using the visual analogue scale. There was no significant difference between the two groups regarding the type of infertility or previous delivery [8]. The results indicated the effect of evening primrose on reducing pain during the insertion of the speculum and the injection of the contrast material during hysterosalpingography.

Alvandipour et al. compared the effect of evening primrose and vitamin E in the treatment of periodic mastalgia. The results indicated that vitamin E and evening primrose extract have similar therapeutic effects for the treatment of breast pain and can be used as therapeutic alternatives [15]. According to the results, evening primrose reduces pain during the insertion of the speculum and the injection of the contrast material. These results were not in good agreement with those of our study. It could be attributed to differences concerning the target population and the method of pain measurement. In this study, the visual analogue scale was used.

Pruthi et al. examined the effect of vitamin E and evening primrose oil on pain control in women with mastalgia. They argued that vitamin E and evening primrose extract have similar therapeutic effects for the treatment of pain in the breast and can be used as therapeutic alternatives [16]. These results were in good agreement with those of our study.

## Conclusion

According to the results, the pain during insertion of the speculum and during contrast medium injection was lower in the evening primrose group compared to placebo. Moreover, there was no difference between the two groups in terms of the length and diameter of the cervix. Therefore, evening primrose is capable of managing pain in hysterosalpingography.

## Acknowledgments

This dissertation was approved with the code IR.ARAKMU.REC.1396.42. The registration code is IRCT2017070520258N52 in the Iranian Center for Clinical Trials. We need to express our sincere gratitude to the Clinical Research Council of Valiasr Hospital and the Deputy of the Arak Medical University Research Center for their assistance in conducting this research.

## Conflict of Interest

The authors confirm that there are no conflicts of interest.

## References

[1] Hassa H, Oge T, Aydin Y, Burkankulu D (2014). Comparison of nonsteroidal anti-inflammatory drugs and misoprostol for pain relief during and after hysterosalpingography: prospective, randomized, controlled trial. *J Minim Invasive Gynecol*.

[2] Wallah E (1972). The uterine factor in infertility. *Fertil steril*.

[3] Lindeman H, Mohr J (1976). Co_2_ hysteroscopy diagnosis and treatment. *Am J Obs Gyn*.

[4] Saini P, Kumar A Pretreatment with hydrotubation in infertility management improves pregnancy rates.

[5] Ryan K, Berkowitz R, Barbieri R (1999). Kistner’s gynecology principals and practice.

[6] Donnez J, Nisolle M (2001). An atlas of operative laparoscopy and hysteroscopy.

[7] Excellence NIfHaC Fertility: assessment and treatment for people with fertility problems.

[8] Szymusik I, Grzechocinka B, Marianowski P, Kacynski B (2014). Factors influencing the severity of pain during hysterosalpingography. *International journal of gynecology & obstetrics*.

[9] Nourbala AA, Tahmasebipour N, Akhoundzadeh S, Khani M, Jamshidi AH, Crocus Sativus L (2004). In the treatment of mild to moderate depression: a double-blind, randomized and placebo controlled trial. *Journal of medicinal planets*.

[10] Akhoundzadeh S, Kashani L, Fotouhi A, Jarvandi S, Mobasheri M, Moein M (2003). Comparison of Lavendula Tincture and Imipramine in the treatment of mild to moderate depression. *Iranian Red Crescent Medical Journal*.

[11] Kiss AK, Derwinska M, Granica S (2011). Quantitative analyss of biologically active polyphenols in evening primrose (Oenothera paradoxa) seeds aqueous extracts. *Journal of Food and Nutrition Sciences*.

[12] Zahradniklova L, Schmidtz S, Sekelyova Z, Sekretar S (2008). Fractionation and identification of some phenolics extracted from evening primrose seed meal. *Czech Journal of Food Science*.

[13] Mansel R, Hughes L (1981). A clinical trial of evening primrose oil in mastalgia. *Br J surg*.

[14] Preece P, Baum M, Mansel R (1982). Importance of mastalgia in operable breast cancer. *Br Med J*.

[15] Alvandipour M, Tayebi P, Alizade noraee R, Khodabakhshi H (2011). Comparison between effect of evening primrose and vitamin E in treatment mastalgia. *J Babol Univ Med Sci*.

[16] Pruthi S, Wahner-Roedler D, Torkelson C, Cha S, Thicke L, Hazelton J (2010). Vitamin E and evening primrose oil for management of cyclical mastalgia: a randomized pilot study. *Altern Med Rev*.

[17] Nikbakht R, Barati M, Sattari A, Mohamadbeigi M, Ziafat M, Sattari S (2016). A comparison Assessment between Diagnostic Value of Hysterosalpingography (HSG) Versus Hysteroscopic Uterine Findings in Infertile Patients who Reffered to IVF Department of Ahvaz Imam Khomeini Hospital During 2010. *Scientific Journal of Ilam University of Medical Sciences*.

